# 2061. High Rates of Viremic HIV, Risky Sexual Behaviors, and Interest in Gender Affirming Hormone Therapy among Transgender Individuals Who Engage in Transactional Sex

**DOI:** 10.1093/ofid/ofac492.1683

**Published:** 2022-12-15

**Authors:** Amelia A Cover, Rahwa Eyasu, Ashley Davis, Omar Harfouch, Emade Ebah, Phyllis Bijole, Catherine Gannon, Grace Garrett, Vivian Wang, Michelle Spikes, F N U Imani, Miriam Jones, Henry Masur, Shyam Kottilil, Sarah Kattakuzhy, Elana S Rosenthal

**Affiliations:** Institute for Human Virology (IHV), University of Maryland School of Medicine, Baltimore, Maryland; Institute for Human Virology (IHV), University of Maryland School of Medicine, Baltimore, Maryland; Institute for Human Virology (IHV), University of Maryland School of Medicine, Baltimore, Maryland; University of Maryland – School of Medicine. Department of Infectious Diseases., Baltimore, Maryland; Institute for Human Virology (IHV), University of Maryland School of Medicine, Baltimore, Maryland; HIPS.org, Washington, District of Columbia; Critical Care Medicine Department, National Institutes of Health (NIH), Washington, District of Columbia; Critical Care Medicine Department, National Institutes of Health (NIH), Washington, District of Columbia; Critical Care Medicine Department, National Institutes of Health (NIH), Washington, District of Columbia; HIPS.org, Washington, District of Columbia; HIPS.org, Washington, District of Columbia; HIPS.org, Washington, District of Columbia; Critical Care Medicine Department, National Institutes of Health (NIH), Washington, District of Columbia; Institute for Human Virology (IHV), University of Maryland School of Medicine, Baltimore, Maryland; Institute for Human Virology (IHV), University of Maryland School of Medicine, Baltimore, Maryland; Institute for Human Virology (IHV), University of Maryland School of Medicine, Baltimore, Maryland

## Abstract

**Background:**

In the U.S., HIV transmission persists largely through sexual transmission in gender and sexual minorities. Transactional sex (TS) is a known risk factor for HIV transmission, yet risk behaviors and engagement in HIV treatment and other medical services among transgender (TG) individuals who have TS are poorly understood.

**Methods:**

PATCH is a natural history study of TG individuals in Washington, DC. Participants complete laboratory testing and surveys, including assessment of TS – defined as previous year sex in exchange for drugs, money or shelter. Fisher’s exact test was used for statistical analysis.

**Results:**

Of 54 participants assigned male at birth (AMAB), 21(39%) endorsed TS, the majority of whom were female (81%), Black (95%), median age 35, and HIV+ (76%; Table 1). Of those with HIV, 12 (75%) were prescribed ART, though only 8 (50%) had HIV VL < 200.

The majority of TG people with TS had sex weekly or more (75%), with > 5 partners/year (62%) and used their penis (80%) and anus (90%) during TS. A minority consistently used condoms in receptive anal sex (29%), penetrative anal sex (25%), penetrative vaginal sex (17%), and chem sex (8%).

While 95% had ever taken gender affirming hormone therapy (GAHT), 62% were not prescribed GAHT at screening, of whom, 54% were interested in seeing a provider for GAHT.

When compared to non-TS participants, TG individuals with TS were more likely to have chem sex (p< 0.01), use drugs daily or more (p=0.04), use amphetamines (p< 0.01) and cocaine (p< 0.01), and less likely to have GAHT prescribed by a provider (p=0.03). HIV+ patients with TS were less likely to have HIV VL< 200 (p=0.02).

**Table 1:**
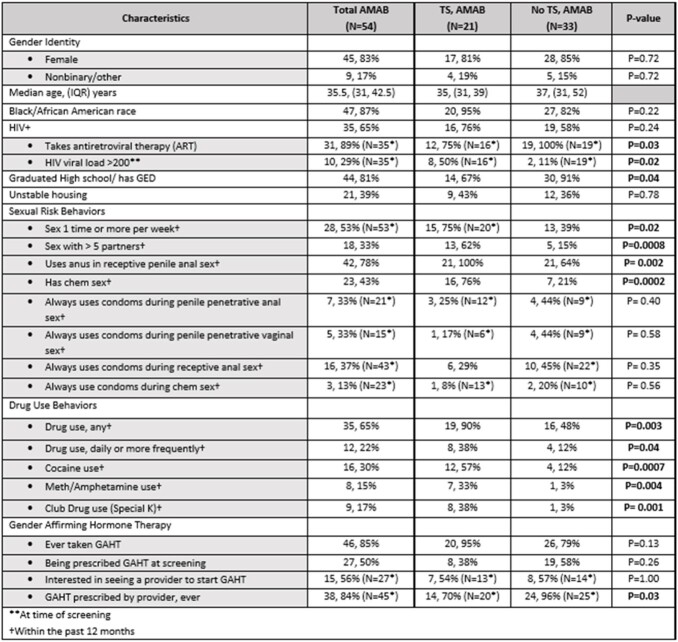
Participant Characteristics and Associations with Transactional Sex

**Conclusion:**

This cohort of AMAB TG individuals with TS had high rates of viremic HIV, and multiple risk factors for HIV transmission, including frequent condomless sex with multiple partners. Additionally, those with TS were more likely to have high risk drug use, indicating that novel strategies to decrease risk associated with chem sex, particularly stimulant use, should be prioritized. Further, nearly all TG participants with TS had taken GAHT, yet were less likely to get GAHT from a provider. As many had interest in seeing a provider for GAHT, co-locating GAHT with HIV treatment and prevention services could help providers engage this population and address HIV-related risks.

**Disclosures:**

**Shyam Kottilil, MD, PhD**, Arbutus Pharmaceuticals: Grant/Research Support|Gilead: Grant/Research Support|Merck: Grant/Research Support|Regeneron Pharmaceuticals: Advisor/Consultant|Silverback Therapeutics: Advisor/Consultant|The Liver Company: Advisor/Consultant|Yufan Biotechnologies: Advisor/Consultant **Sarah Kattakuzhy, MD**, Gilead: Grant/Research Support **Elana S. Rosenthal, MD**, Gilead: Grant/Research Support|Merck: Grant/Research Support.

